# Seminal Vesicle Treatment for Localized Prostate Cancer Treated with External Beam Radiotherapy

**DOI:** 10.3390/curroncol30070483

**Published:** 2023-07-10

**Authors:** Tanner Steed, Nikki Chopra, Jihyun Yun, Jordan Hill, Benjamin Burke, Sunita Ghosh, Brad Warkentin, Nawaid Usmani

**Affiliations:** 1 Department of Oncology, University of Alberta, 11560 University Ave, Edmonton, AB T6G 1Z2, Canada; tanner.steed@albertahealthservices.ca (T.S.); nikki.chopra@albertahealthservices.ca (N.C.); jihyun.yun@albertahealthservices.ca (J.Y.); jordan.hill@albertahealthservices.ca (J.H.); ben.burke@albertahealthservices.ca (B.B.); sunita.ghosh@albertahealthservices.ca (S.G.); brad.warkentin@albertahealthservices.ca (B.W.); 2 Department of Oncology, Faculty of Medicine and Dentistry, University of Alberta, 11560 University Ave, Edmonton, AB T6G 1Z2, Canada

**Keywords:** prostate, radiotherapy, seminal vesicle, contour

## Abstract

This study retrospectively reviewed data from men with localized prostate cancer treated with external beam radiotherapy (EBRT). We identified 359 men with localized prostate cancer treated with curative EBRT at the Cross Cancer Institute between 2010–2011. The volume of seminal vesicles (SVs) treated as well as dose values were extracted. These volumes were compared to gold standard contours drawn by a trained expert based on consensus European Society for Radiotherapy and Oncology (ESTRO) contouring guidelines. Patient and tumor characteristics were extracted for these patients. Memorial Sloan Kettering prostate cancer nomogram was used to assign a predicted risk of SV involvement for each patient based on baseline tumor characteristics. In patients with a predicted risk of SV involvement greater than 15% (*n* = 184), 86.5% (SD = 18.6) of the base of the SVs were treated with EBRT, compared to 66.7% (SD = 32.6) for patients with a predicted risk of SV involvement less than 15% (*n* = 175, *p* < 0.0001). Similarly, the mean percentage of proximal and total SV volumes treated with EBRT was 75.6% (SD = 24.4) and 68.7% (SD = 26.0) for patients with a predicted risk of SV involvement of greater than 15%, compared to 50.3% (SD = 31.0, *p* < 0.0001) and 41.0% (SD = 27.8, *p* < 0.0001) for patients with a risk of less than 15%. The results indicate that all parts of the SVs are more likely to be contoured in men with >15% risk of SV involvement than those with <15% risk. However, radiation oncologists still contour a high percentage of SVs in men with <15% risk of SV involvement, suggesting that there may be over-treatment of SVs that increases the risk of rectal or bladder toxicity.

## 1. Introduction

Radiotherapy is a common treatment modality for men with localized prostate cancer, with recent technological advances allowing for improved image guidance and conformal treatments. While the entire prostate gland has historically been treated for patients undergoing definitive radiotherapy, there has been significant variation in whether or not to include the seminal vesicles in target volumes, and if so, what volume of the seminal vesicles is treated.

Historically, radiation oncologists utilized surgical data to identify the risk of microscopic disease involvement in designing radiation target volumes. A detailed pathologic analysis by Kestin et al. on 344 radical prostatectomy specimens is often referenced for identifying the pathologic risk of seminal vesicle involvement [1]. Their study found that in patients without any risk factors (PSA over 10, Gleason over 6, T-stage over T2a), only 1% had seminal vesicle (SV) involvement; 15% of patients with one risk factor had SV invasion; in patients with all three risk factors, 58% had SV invasion [2]. Similar findings were reported in the British Journal of Urology in 2013.

Based on these and other similar reports, a literature review published in 2007 [3] recommended that seminal vesicles should be excluded from the clinical target volume (CTV) in patients who have a prostate-specific antigen (PSA) of less than 10, a Gleason score of 6 or less, a clinical stage of equal to or less than T2a, and less than 50% of biopsy cores being positive, as these patients had less than a 5% risk of SV invasion. They also demonstrated that the spread of cancer from the prostate to the seminal vesicles is continuous, and the initial invasion of the seminal vesicles is most frequent in the part proximal to the prostate. By including only the prostate in the CTV, the proximal half of the seminal vesicles ended up getting an adequate dose of radiation to cover microscopic diseases in many cases. In patients with one or more of the previously mentioned risk factors, they suggested including the seminal vesicles but could not make a strong recommendation as to what volume or length to treat.

When treating the seminal vesicles, there is no agreed upon standard of what volume or length should be included. The previously mentioned study by Kestin et al. [1] showed that when SV involvement was noted, the median length of involvement was 1 cm. In high-risk patients, only 4% of patients had SV involvement greater than 2 cm. In light of this and other pathologic studies, previous EORTC guidelines [4] suggested that in high-risk prostate cancer, the proximal 2 cm of SV be included in the CTV and the proximal 1 cm in intermediate-risk patients. A single-center retrospective study was published in 2014 [5] that examined 134 consecutive patients. These patients’ SVs were contoured using computed tomography (CT) images both according to actual anatomy and according to the EORTC guidelines from that time. They found that in many cases the anatomic 1 or 2 cm proximal part of the SV was being missed when contoured according to the EORTC guidelines and suggested instead a 1.4 cm and 2.2 cm delineation of SV in the axial plane to adequately cover the actual 1 and 2 cm of the anatomy of the SV.

In 2018, ESTRO released and updated a consensus guideline for computed tomography/magnetic resonance imaging (CT/MRI) target delineation in localized prostate cancer [4]. In these guidelines, they suggest for low-risk prostate cancer (according to the National Comprehensive cancer network [NCCN] risk stratification) the seminal vesicles should not be included. For intermediate prostate cancer, at least the proximal 1.4 cm of SV in the axial plane should be included. And for high-risk, at least 2.2 cm of the proximal SV in the axial plane should be included. The ESTRO consensus guidelines suggest that for high-risk patients, a low dose volume should cover the pelvic lymph nodes and a high dose volume to cover the prostate. However, it does not specify the dose to which the seminal vesicles should be treated.

The ESTRO consensus guidelines currently represent the most up-to-date guidelines on how and when to contour the seminal vesicles. However, anecdotal experience suggests that these guidelines are not universally followed and there is wide variation in how different physicians approach SV delineation on CT imaging and dosing of these volumes. Since including the seminal vesicles in the CTV increases the dose to both the rectal and bladder, it is in the patient’s best interest to find the optimal point of balancing between adequate coverage and over-treatment. In this study, we aim to examine what is currently being contoured by practicing radiation oncologists and how this correlates to existing consensus guidelines. In future studies, we hope to examine how the inclusion of the seminal vesicles affects patient outcomes.

## 2. Materials and Methods

We identified 359 consecutive patients who were treated with definitive, intensity- modulated radiotherapy (IMRT) at the Cross Cancer Institute in Edmonton, Alberta from 2010–2011. All patients were over 18 and had a pathologically proven diagnosis of prostate adenocarcinoma and were receiving radiotherapy as their primary treatment. Only patients who were treated with standard fractionation (1.8–2.0 Gy per fraction) were included. Patients were allowed to receive androgen deprivation therapy in this study. A chart review of this cohort was performed using both physical charts and electronic medical record data to extract patient and tumor characteristics (Table 1).

The study was approved by the Health Research Ethics Board of Alberta (HREBA.CC-20-0502). Treatment planning and calculation of dose metrics were performed using Varian’s Eclipse software. Values for the dose delivered were extracted from Varian’s Eclipse software. These patients were originally treated by 10 different radiation oncologists in Edmonton, Alberta. In 2010, the individual oncologists in this group had been independently practicing between 0 to 24 years, with a mean value of 9.6 years [6]. All patients had prior CT simulation scans, performed with patients in the supine position with an empty rectum, full bladder, and a slice thickness of 3 mm.

**Figure 1:**
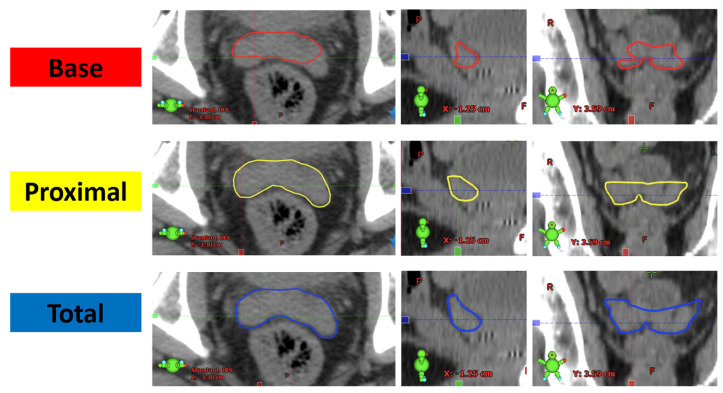
Gold-standard volumes for SV base, proximal, and total according to current ESTRO guidelines.

Three different volumes were contoured, the SV base, SV proximal, and SV total (Figure 1). SV base included all of the SV within a 1 cm radial expansion of the prostate contour. This volume is an SV contour based on the volume of SVs included in the PROFIT trial, for which our centre accrued patients [7]. SV proximal included any SV within a 1.4 cm distance in the axial plane from the most inferior slice of SV. SV total included any SV within a 2.2 cm distance in the axial plane from the most inferior slice of SV. SV proximal and total were contoured according to the ESTRO consensus guidelines. These three volumes were considered the gold standard, and software was used to compare them to the volume of SV within the CTV that had been originally treated. The gold standard contours were completed by both residents and medical students and were reviewed by staff radiation oncologists at the Cross Cancer Center. Those involved in contouring these gold standard volumes had in-depth training regarding how to contour accurately. This was followed by a probationary period, where every contour completed would be reviewed by a staff oncologist. Once consistently contouring correctly, they would continue to have a sampling of patient contours reviewed to ensure accuracy.

When calculating the dose delivered to the gold standard SV volumes, patients were only included when there was an intent to treat the seminal vesicles. This intent to treat was defined as having at least 80% overlap between the gold standard SV volume and the originally treated clinical CTV. The gold standard contours were created in copies of the original clinical treatment plans for analysis. This required recalculation of the dose distributions using current versions of the Eclipse dose calculation (AAA) and leaf motion calculator algorithms since the original algorithms were no longer active in our treatment planning system. Minimum, mean, and maximum doses for the SV volumes of each patient were extracted from these treatment plans.

Mean and standard deviations were reported for normally distributed continuous variables, and median and range were reported for non-normally distributed continuous variables. Frequency and proportions were reported for the categorical variables. Chi-square tests were used to compare the proportions of two categorical variables. Independent *t*-tests were used to compare the mean between the two groups.

Our analyses were stratified based on either a predicted risk of SV invasion being over or under 15% or according to the National Comprehensive Cancer Network risk stratification groups [8]. The NCCN stratifies patients into either very low-risk, low-risk, intermediate-risk, high-risk, or very-high-risk prostate cancer. This is based on the patients clinical T-stage, Gleason score, pre-treatment PSA value, and amount of involved cores present. For this study, we have combined the very low-risk and low-risk groups into a single group we refer to as low-risk. We also combined the very-high-risk group and high-risk group into a single group we refer to as high-risk. To the predict risk of SV invasion being over or under 15%, we used patient demographic data and the Memorial Sloan Kettering pre-radical prostatectomy nomogram [9] to assign a value based on available data. The Memorial Sloan Kettering Cancer Center (MSKCC) pre-radical prostatectomy nomogram is a tool for patients diagnosed with prostate cancer and their physicians who have not yet begun treatment. This nomogram predicts the extent of cancer and long-term results following radical prostatectomy (surgery to remove the prostate gland and surrounding lymph nodes). Using dynamic statistical formulas, this nomogram draws on data from more than 10,000 prostate cancer patients treated at Memorial Sloan Kettering. The nomogram helps to predict the probability of lymph node and seminal vesicle involvement based on the patient’s age, PSA level, Gleason pattern, clinical tumor stage, and biopsy cores [9].

All statistical analysis was conducted using SPSS (IBM Corp. Released 2017. IBM SPSS Statistics for Windows, Version 25.0. Armonk, NY, USA: IBM Corp.) version 25 software. A *p*-value < 0.05 was used for statistical significance.

## 3. Results

Of the 359 patients included in this study, 184 were found to have over a 15% risk of SV invasion and 175 were found to have equal to or less than a 15% risk, according to the MSKCC nomogram. As demonstrated in Table 1, the nomogram assigns patients to the over 15% risk group who tended to be older, have more advanced T-stage, belong to a higher Gleason grade group, and have a higher PSA value.

In patients with a predicted risk of SV involvement greater than 15%, 86.5% of the gold standard SV base volume was treated, compared to 66.7% for patients with a predicted risk of SV involvement less than 15% (Table 2, *p* < 0.0001). For the proximal SV volume, 75.6% of the gold standard volume was treated in the over 15% risk group while only 50.3% was treated in the under 15% risk group (*p* < 0.0001). For the total SV volume, 68.7% of the gold standard volume was treated in the over 15% risk group while 41.0% was treated in the under 15% risk group (*p* < 0.0001).

When stratifying patients according to NCCN guidelines, the percentage of SV base gold-standard volume treated was 48.9% in the low-risk group, 65.7% in favorable intermediate risk, 81.6% in unfavorable intermediate-risk, and 86.4% in high-risk patients (Table 3, *p* < 0.0001). For the SV proximal volume, 33.1% of the gold-standard volume was treated in low-risk patients, 47.0% in favorable intermediate-risk, 64.0% in unfavorable intermediate-risk, and 78.2% in high-risk patients (*p* < 0.0001). For the SV total volume, 24.6% of the gold-standard volume was treated in low-risk patients, 37.3% in favorable intermediate-risk patients, 53.5% in unfavorable intermediate-risk patients, and 72.7% in high-risk patients (*p* < 0.0001).

For patients for whom there was an intent to treat seminal vesicles, SV base volumes were treated to a higher dose compared to SV proximal or total, with SV total being treated to the lowest average dose (Table 4). The average of the minimum dose was 7664 cGy to the SV base volume, 7325 cGy to the SV proximal volume, and 6986 cGy to the SV total volume. A similar trend is seen for the maximum and mean doses. The standard deviation for the minimum dose delivered to the SV total volume is 882, which is larger than the standard deviation for the minimum dose delivered to the SV proximal (SD = 598) or SV base (SD = 272) volumes.

## 4. Discussion

SV invasion is seen more commonly in higher-risk groups of prostate cancer. SV invasion is correlated with higher rates of biochemical recurrence and lower rates of overall survival in both those treated with prostatectomy alone, as well as those treated with prostatectomy followed by adjuvant radiation [10]. We also see that treating the seminal vesicles with adjuvant radiation significantly improved outcomes compared to those who were treated with prostatectomy alone [10]. We can conclude that it is important to include the SV in the CTV in select cases. However, including the SV in the CTV also increases radiation dose to a patient’s rectum and bladder, which can cause both short- and long-term toxicity; thus, over-treatment should be avoided. The decision about when to include the SV in the CTV and to what extent can be challenging and is made on a case-by-case basis at the discretion of the treating physician.

There is no universally agreed-upon standard of how to treat SV, and this question has never been studied in a prospective trial. The EORTC consensus guidelines provide an evidence-based approach to the treatment of prostate cancer, but anecdotal experience suggests that physicians often treat according to institutional standards or in accordance with the way they were trained before current guidelines.

In this study, we examined how treating physicians at a single institution treated and delineated SVs compared to current ESTRO guidelines. According to ESTRO guidelines, patients with low-risk prostate cancer should have had no coverage of any part of the SVs. For intermediate-risk prostate cancer, guidelines would suggest 100% coverage of the SV proximal volume. For high-risk prostate cancer, guidelines would suggest 100% coverage of the SV total volume. Current guidelines do not use our other stratification method of over/under 15% risk of SV invasion obtained from the MSKCC prostate cancer nomogram. However, intermediate and high-risk prostate cancer typically have greater than a 15% risk of SV invasion according to previous pathological studies [11]. Based on this evidence, it would be expected that those in the over 15% risk group should have at least the SV proximal included in the treatment area, and those in the under or equal to 15% risk group should not require any coverage of SVs.

In our data, the under 15% risk group shows significant amounts of SV being treated with an average of 41% of the total SV being covered. For the over 15% risk group, we see that on average significantly less than the recommended SV volumes are being treated, as 76% of the proximal SV volume is treated, and 69% of the total SV volume is treated.

Similar findings are seen in the patients when stratified by NCCN risk groups. On average, in low-risk patients, a significant volume of SV is being treated with 25% of the total SV volume being covered. In intermediate and high-risk patients, significantly less than recommended volumes of the proximal and total SV volumes are being treated with 47% of proximal SV being covered in favorable intermediate risk, 64% of proximal SV being covered in unfavorable intermediate risk, and 73% of SV total being covered in high-risk patients.

In the low-risk group and under 15% SV invasion risk group, there is no evidence or consensus that any amount of the SVs should be treated. When the SVs of these patients are included, the patients are exposed to an increased risk of radiation toxicity without any evidence of benefit. When looking at the high or intermediate-risk groups, and the over 15% SV invasion risk group, we note these patients are having their SV treated less than what is recommended by current guidelines. There is evidence that outcomes are worse when prostate cancer invades the SVs [10] and by under-treating these patients they may have a higher risk of biochemical recurrence and worse overall survival.

On average, SV base volumes were treated to a higher dose while SV total volumes were treated to a lower dose. This may seem counter-intuitive at first, as the SV total volume was rarely treated in low-risk patients, and one could expect higher doses used in higher-risk patients. However, this likely represents further variation in how physicians are treating seminal vesicles. Our data suggests that in a low or intermediate-risk patient, the prostate volume is likely just expanded slightly to cover some or all of the seminal vesicles and this volume is treated to the same dose as the prostate. In high-risk patients, where there is a high and low dose CTV, it seems that some physicians will treat the SV as part of the low dose elective nodal volume, while others will treat them as part of the high dose prostate volume. This is further reflected in the larger standard deviation and dose ranges seen in the SV total group in Table 4.

We recognize some limitations of our study. This is a single-institution retrospective analysis and other centers may treat it differently. These patients were also treated in 2010–2011, and at that time the consensus guidelines suggested a smaller (1 and 2 cm SV coverage instead of current standards of 1.4 and 2.2 cm) volume of SV be treated. There is also an element of subjectivity when delineating and defining seminal vesicles on a CT image. Our study has not taken into account daily variation in the position of the SVs or set-up errors, as we have looked only at CTVs and not PTVs. These volumes do not take into account the addition of ADT, which may also alter target volumes. Current risk estimates of seminal vesicle invasion are based on a handful of clinical and pathological factors and in the future the addition of other imaging (i.e., MRI and PSMA PET), pathological or novel biochemical tools may improve the accuracy of these estimates. There is also an element of contouring variability, both in a case-by-case setting for a given radiation oncologist and also between different radiation oncologists.

Although some limitations exist, this study represents the best practice of 10 different radiation oncologists and has a patient population large enough that we can be confident in statistically significant differences between the groups. Our findings may lead to future avenues of study, namely investigating how SVs are treated at other centers and if there have been changes to how SV is treated now versus 10–11 years ago. It is also worth noting that while we have shown these patients are not being treated according to current guidelines, these are consensus guidelines and not based on prospective outcome-related data. One may consider if the way patients are being treated may be just as good or better than what the consensus guidelines suggest. In addition to the lack of evidence regarding the correct SV volumes, there continues to be uncertainty on the optimal dose required to treat microscopic disease in the seminal vesicles. In the future, we plan to analyze long-term data for these patients post-treatment and determine the time to biochemical recurrence. With this data, we will retrospectively analyze if there is a correlation between the amount of SV coverage in intermediate and high-risk patients, as well as the dose delivered to different regions of the SVs, and the time to biochemical recurrence.

## 5. Conclusions

Our results show that current practice by radiation oncologists may not be in line with the most up-to-date consensus guidelines for prostate cancer radiotherapy, specifically in relation to how and when to treat SVs. Of the patients analyzed, those with low-risk prostate cancer have more of the SV volume treated than recommended, while those with intermediate or high-risk prostate cancer have less SV volume treated than recommended by current guidelines. This may be exposing overtreated low-risk patients to unnecessary toxicity, while also causing increased rates of biochemical recurrence and worse overall outcomes in undertreated intermediate and high-risk patients. We also found that there is significant variation in the doses being used to treat different SV volumes. Further study will be required to determine if the differences in SV volume treated and the doses are correlated with any difference in patient outcomes.

## Figures and Tables

**Table 1:** Patient characteristics according to predicted risk of SV invasion greater or less than 15%.

Variables		>15% (*n* = 184)	≤15% (*n* = 175)	*p*-Value	Overall (*n* = 359)
**Age**				0.017	
	Mean	68.9	67.2		68.0
	Range	49–99	40–99		40–99
**T-stage**				<0.0001	
	T1	22 (12.0)	81 (46.3)		103 (28.7)
	T2	130 (70.7)	91 (52.0)		221 (61.6)
	T3	31 (16.8)	3 (1.7)		34 (9.5)
	T4	1 (0.5)	0 (0)		1 (0.3)
**Gleason Grade Group**				<0.0001	
	1	1(0.5)	97 (55.4)		98 (27.3)
	2	58 (31.5)	67 (38.3)		125 (34.8)
	3	58 (31.5)	7 (4.0)		65 (18.1)
	4	41 (22.3)	4 (2.3)		45 (12.5)
	5	26 (14.1)	0 (0)		26 (7.2)
**PSA**				<0.0001	
	Mean	23.5	9.7		16.8
	Range	0.6–100.0	0.6–71.2		0.6–100.0
**Risk Category**				<0.0001	
	Low	0 (0)	53 (30.3)		53 (14.8)
	F. Intermediate	0 (0)	39 (22.3)		39 (10.9)
	U. Intermediate	58 (31.5)	68 (38.9)		126 (35.1)
	High	126 (68.5)	165(8.6)		141 (39.3)
**Prescribed Number of Fractions**				0.104	
	Mean	37.6	37.6		37.6
	Range	35–41	36–39		35–41

**Table 2:** Seminal vesicle volumes treated stratified by predicted risk of SV invasion greater or less than 15%.

	%SV Base Treated	% SV Proximal Treated	% SV Total Treated
**SV ≤ 15% (*n* = 175)**	66.7	50.3	41.0
	(SD = 32.6)	(SD = 31.0)	(SD = 27.8)
**SV > 15% (*n* = 184)**	86.5	75.6	68.7
	(SD = 18.6)	(SD = 24.4)	(SD = 26.0)
***p*** **-value**	<0.0001	<0.0001	<0.0001

**Table 3:** Seminal vesicle volumes treated stratified by NCCN risk groups.

	% SV Base Treated	% SV Proximal Treated	% SV Total Treated
**Low risk (*n* = 52)**	48.9	33.1	24.6
	(SD = 32.7)	(SD = 29.5)	(SD = 21.9)
**Favorable intermediate risk (*n* = 37)**	65.7	47.0	37.3
	(SD = 33.4)	(SD = 26.3)	(SD = 21.9)
**Un. intermediate risk (*n*= 122)**	81.6	64.0	53.5
	(SD = 23.7)	(SD = 26.8)	(SD = 25.9)
**High risk (*n* = 138)**	86.4	78.2	72.7
	(SD = 19.3)	(SD = 23.8)	(SD = 25.7)
***p*** **-value**	<0.0001	<0.0001	<0.0001

**Table 4:** Dose delivered to seminal vesicle volumes.

	Min Dose (cGy)	Max Dose (cGy)	Mean Dose (cGy)
**SV base (*n* = 244)**	7664	8021	7885
	(SD = 272)	(SD = 256)	(SD = 239)
	(Range = 6243–8321)	(Range = 6960–8694)	(Range = 6515–8506)
**SV proximal (*n* = 153)**	7325	8034	7822
	(SD = 598)	(SD = 286)	(SD = 312)
	(Range = 5393–8321)	(Range = 7128–8731)	(Range = 6559–8510)
**SV total (*n* = 106)**	6986	8028	7735
	(SD = 882)	(SD = 308)	(SD = 429)
	(Range = 4466–8321)	(Range = 7194–8731)	(Range = 6447–8508)
***p*** **-value**	<0.001	0.903	<0.001

## Data Availability

Research data are stored in an institutional repository and will be shared upon request to the corresponding author.
